# Juvenile dermatomyositis in a 4‐year‐old Kenyan girl

**DOI:** 10.1002/ccr3.816

**Published:** 2017-01-17

**Authors:** Marlous L. Grijsen, Deborah Mchaile, Inge Geut, Raimos Olomi, Maitseo Nwako, Luis Requena, William P. Howlett, Daudi R. Mavura, Marieke C. J. Dekker

**Affiliations:** ^1^ Department of Dermatology Leiden University Medical Center Leiden The Netherlands; ^2^ Regional Dermatology Training Centre Kilimanjaro Christian Medical Centre Moshi United Republic of Tanzania; ^3^ Department of Pediatrics Kilimanjaro Christian Medical Centre Moshi United Republic of Tanzania; ^4^ Department of Neurology Medisch Spectrum Twente Enschede The Netherlands; ^5^ Department of Internal Medicine Kilimanjaro Christian Medical Centre Moshi United Republic of Tanzania; ^6^ Department of Dermatology Fundación Jiménez Díaz Universidad Autónoma Madrid Spain

**Keywords:** Africa, calcinosis cutis, juvenile dermatomyositis, Tanzania

## Abstract

To our knowledge, this is the first case report of juvenile dermatomyositis (JDM) in Tanzania. It demonstrates that the characteristic cutaneous findings of JDM may easily be overlooked, especially on dark skin, and the difficulty of clinical management in resource‐constrained settings.

## Graphical abstract

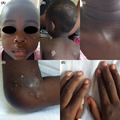

## Introduction

Juvenile dermatomyositis (JDM) is a rare systemic autoimmune disease in children affecting primarily the sun‐exposed skin, resulting in a distinctive cutaneous eruption, and the proximal musculature. The age of onset is between 2 and 13 years with 20% of patients presenting before the age of 4 years. As opposed to adult dermatomyositis, children present more often with small vessel vasculopathy and inflammation plus necrosis of the muscles. In addition, myositis‐specific antibodies are less frequently present, malignancies are sporadic, the mortality rate is lower, and cutaneous calcifications are common (up to 40%) 1. To date, only few cases of JDM have been reported in sub‐Saharan Africa 2, 3, 4, 5 of which none from East Africa. We describe a 4‐year‐old Kenyan girl who presented with calcinosis cutis most likely secondary to JDM causing contractures.

## Case Presentation

A 4‐year‐old, HIV‐negative, Kenyan girl was admitted to the Paediatric Ward of the Kilimanjaro Christian Medical Centre (KCMC) in Moshi, Tanzania, with an 8‐month history of hard and painful, non‐itchy nodules of the skin of which some had ulcerated. The nodules had started around her knees and elbows and had spread to the shoulders. She had a dry cough since 2 weeks, general malaise, a low‐grade fever, weight loss since a few months and was unable to walk. There was no history of night sweats or exposure to tuberculosis. Otherwise, she had a normal development and was the sixth child in a healthy, nonconsanguineous family. She had already attended several hospitals in her region, but no cause for the complaints was found.

**Figure 1:**
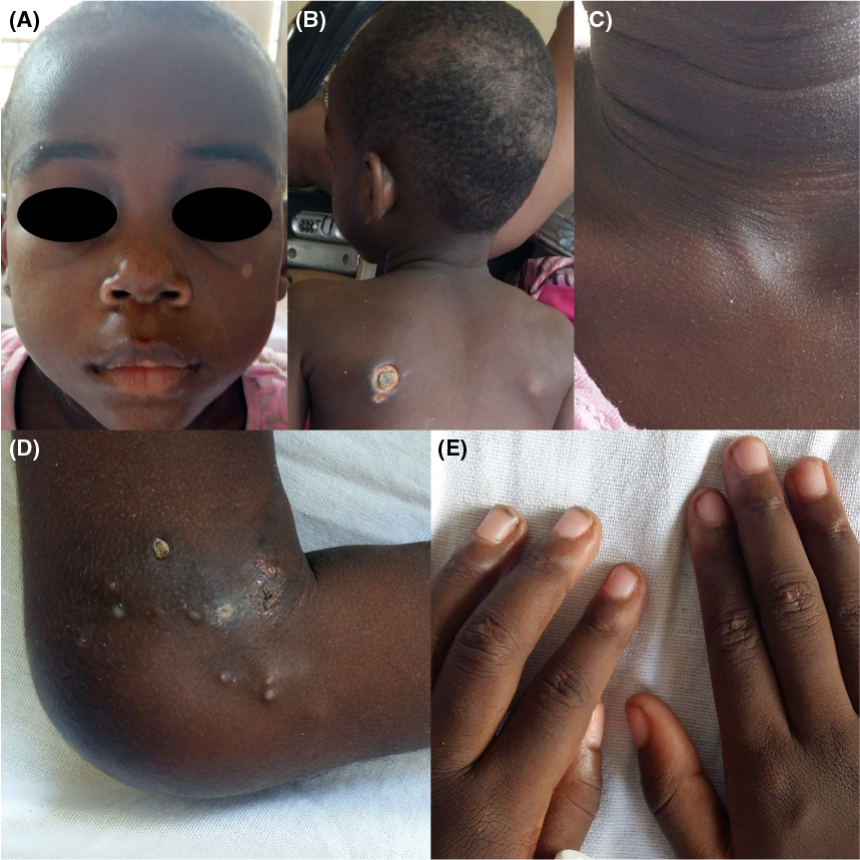
Edema with hyperpigmentation above the upper eyelids (heliotrope rash, A); ecthyma‐like ulcer on the left shoulder and nonscarring alopecia on the scalp (B); hyperpigmented rash in the neck (C); calcified papules and nodules around the knee of which some have expelled calcified material (D); small flat‐topped papules over the proximal and distal interphalangeal joints (possible early establishment of Gottron's papules, (E)

At physical examination, we saw an afebrile, ill‐looking and wasted child (length 1.03 m, weight 10 kg, BMI 9.4 kg/m^2^). On the shoulders, elbows, and knees and along the fascia of several proximal muscles, including hamstrings, quadriceps, abdominal, and trapezius muscles, multiple hard and calcified papules and nodules were observed varying in size from 5 to 15 mm. Some of these lesions expelled calcified material leaving ecthyma‐like ulcers (Figure 1B, D). Musculoskeletal inspection showed mild contractures in knees, elbows and shoulder joints. Upon neurological examination, the muscle bulk was in proportion with her poor nutritional status, but not reminiscent of atrophy, as various limb girdle muscles appeared still fairly normal and strong when tested supine. However, she was not able to get up from the supine position, elevate both her arms, or comb her hair in the seated position. Gower's sign was negative. Bedside testing did not reveal outspoken muscle weakness, Medical Research Council scale was 4/5. However, pain may have interfered with maximal contraction. There was no distal weakness or atrophy. Reflexes and sensation were unremarkable.

The dermatologist was consulted, and it was noticed that the child had slight edema with hyperpigmentation above the upper eyelids (heliotrope rash), small flat‐topped skin‐colored papules over the proximal and distal interphalangeal joints (possible early establishment of Gottron's papules), a hyperpigmented rash in her neck, and a nonscarring alopecia (Figure 1A–C, E). There were no further abnormalities of the skin, nails, or oral cavity.

**Figure 2:**
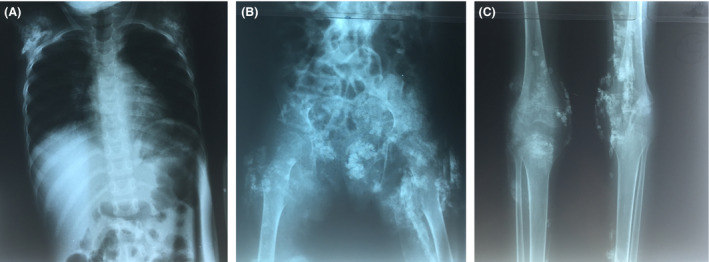
X‐ray of chest (A), hips (B), and knees (C) demonstrating numerous calcifications in joints, muscles, and subcutaneous tissues.

Laboratory examinations revealed increased serum levels of lactate dehydrogenase (LDH, 671 [reference value 240–480] U/l), alanine transaminase (ALT, 39 [2–33] U/l), and aspartate aminotransferase (AST, 60 [2–31] U/l). Serum creatine kinase (CK) was within the normal range (158 [26–192] U/l). Full blood count, kidney function, and serum phosphate and calcium levels were normal. Erythrocyte sedimentation rate (ESR) was raised (70 [3–13] mm/h). X‐rays of chest, hips, and legs showed numerous calcifications peri‐articular and in the soft tissues (Figure 2). Chest X‐ray demonstrated parenchymatous infiltrates compatible with pneumonia (likely due to aspiration in this bedridden child), but no evidence of pulmonary tuberculosis. Skin biopsy from a nodule on the knee revealed deposits of calcium in the dermis confirming the diagnosis of calcinosis cutis. No other histopathological abnormalities were seen. We were not able to perform (myositis‐specific) autoantibodies, for example, ANA and anti‐Mi2, a muscle biopsy, electromyography, or MRI as these examinations at the time were not available at KCMC. Based on the clinical, laboratory, and histopathological findings, the diagnosis of JDM was made.

The patient was started on oral prednisolone and broadspectrum antibiotics (ceftriaxone and metronidazole), and physiotherapy was initiated. She showed mild clinical improvement, but then disappeared from the hospital ward a few days after initiation of treatment.

## Discussion

JDM is a systemic autoimmune vasculopathy characterized by muscle weakness and distinctive skin findings. The diagnostic criteria for JDM, as proposed by Bohan and Peter, are included in Table 1 6. JDM is diagnosed if four of these criteria are present, including the skin rash; the diagnosis is probable in the presence of two criteria in addition to the skin rash. Due to limited resources, we did not perform a muscle biopsy, electromyography, or MRI to support our clinical impression. The proximal muscle weakness could not be reliably assessed due to concomitant pain and contractures, which limited range of movement. This means that although muscle weakness could not be directly demonstrated, it may very well have been present. Hence, it should be noted that the presented case does not fully comply with the diagnostic criteria for JDM. Yet, to diagnose JDM in daily practice on the basis of these criteria is challenging, especially in resource‐limited settings but also in young children, as both electromyography and muscle biopsy are invasive. Recently, an international survey among pediatric rheumatologists demonstrated that proximal muscle weakness, specific skin features, and raised muscle enzymes were the most frequently used criteria for identifying JDM. EMG and muscle biopsy were used as diagnostic support in only 56 and 61% of patients, respectively 7. Nowadays, MRI is increasingly replacing the latter investigations, but is not always available in low‐ and middle‐income countries. In our patient, the combination of probable proximal muscle weakness, the rise of some of the muscle enzymes (LDH, ALT, AST) and an elevated ESR, extensive calcinosis cutis and minimal, but distinctive cutaneous lesions (heliotrope rash, possible Gottron's papules, skin ulcerations, and alopecia) make the diagnosis of JDM very likely.

**Table 1:** Diagnostic criteria of juvenile dermatomyositis as proposed by Bohan and Peter 6

Presence of at least one of the characteristic skin rashes^a^, in addition to three of the following criteria
Symmetrical proximal muscle weakness
Elevated muscle enzymes (CK, LDH, ALT, AST, aldolase)
Electromyographic changes compatible with myopathy
Muscle biopsy showing necrosis and inflammation

a For example, heliotrope rash, Shawl's sign, or Gottron's papules.

Dystrophic calcification is a well‐recognized complication of JDM and involves the deposition of insoluble calcium salts in the cutaneous and subcutaneous tissues. Patients may present with firm to rock‐hard irregular nodules or with larger subcutaneous plaques along the fascia or in the intramuscular connective tissue causing extensive areas of calcification, leading to functional impairment. Elbows, knees, buttocks, and shoulders are typically affected. The calcified lesions may ulcerate and discharge a white, chalky material causing ecthyma‐like lesions. Calcinosis is thought to be a consequence of prolonged inflammation and generally develops 2–3 years after disease onset 8. Our patient presented with widespread calcifications in the skin and muscles, which reflects delay in treatment of an active myositis, which is not unusual in a setting like East Africa and may be explained by financial constraints, lack of access to appropriate care, and poor health education. However, it may also be explained by JDM‐specific autoantibodies like nuclear matrix protein 2 (NXP2) that is associated with calcinosis and severe myositis 9. A recent retrospective study from South Africa showed that African children with JDM had high levels of dystrophic calcification and low levels of muscle enzymes, particularly CK, suggesting low‐grade chronic inflammation 2.

Muscle‐derived enzymes, of which CK is most commonly used, are often elevated in JDM indicating muscle damage. In some cases, however, CK may be normal, especially in the early and chronic stage of the disease. In one study, one‐third of the affected children with active disease had a normal serum CK 1, 10. The South African study revealed that the muscle enzymes, specifically CK, were significantly lower in children with calcinosis compared to those without calcifications 2. Another study demonstrated that in untreated JDM after 4.7 months, the different muscle enzymes tended to be normal 11. These studies highlight the importance of not merely relying on CK, but to determine other myositis‐associated enzymes, including transaminases, LDH, and aldolase, as well. Additionally, it shows that as time without therapy increases, the muscle enzymes may normalize. In our case, the CK was normal, but LDH and transaminases were raised. Serum aldolase was not available.

In our differential diagnosis, we considered primary tumoral calcinosis, which is a hereditary disease, has been described in patients of African descent, and is more common in (sub)tropical regions 12, 13. However, we deemed this to be less likely as tumoral calcinosis generally occurs in peri‐articular soft tissues around hip, shoulders, buttocks, and elbow joints and not along the muscles/facies as in our case. Additionally, our patient did not have an imbalance in calcium and phosphate homeostasis, which sometimes is the case in tumoral calcinosis, and had a negative family history despite having five siblings. Finally, tumoral calcinosis does not explain the constitutional and cutaneous symptoms and signs, and raised muscle enzymes. Alternatively, we considered juvenile systemic sclerosis (JSSc) as this may present with muscle weakness and calcinosis cutis as well, and could explain the abnormal pulmonary findings as autoimmune pulmonary involvement. The majority of children with JSSc, however, present with symmetrical hardening/thickening of the skin and/or Raynaud phenomenon, which was not the case in our child. Also, interstitial lung disease has been reported as a complication of JDM 14. Nevertheless, without specific autoantibody testing, it is not possible to completely rule out JSSc.

To our knowledge, this is the first report of JDM in East Africa. The present case demonstrates that the characteristic cutaneous findings of JDM may easily be overlooked, especially on the black skin. It shows the difficulty of clinical management and the importance of a multidisciplinary approach in complex patients, especially in settings where diagnostic means are limited.

## Consent

Written informed consent was obtained from the patient's mother allowing us to publish the photographs and the medical information.

## Authorship

All authors were involved in the treatment and follow‐up of the patient. MLG: drafted the manuscript. MCJD: critically revised the manuscript. All authors reviewed and approved the final manuscript and gave consent for publication. The authors wish to thank Dr. Ben Naafs (dermato‐venereologist Foundation Global Dermatology, the Netherlands) and Samson K. Kiprono (dermato‐venereologist at Moi University in Eldoret, Kenya) for helping to establish the diagnosis and for their useful comments on the manuscript.

## Conflict of Interest

None declared.

## References

[1] Paller, A. S. , A. J. Mancini . 2006. Collagen vascular disorders. Pp. 584–590. Hurwitz clinical pediatric dermatology: a textbook of skin disorders of childhood and adolescence. 3rd edition. W.B. Elsevier Saunders, Philadelphia.

[2] Faller, G. , B. J. Mistry , and M. Tikly . 2014. Juvenile dermatomyositis in South African children is characterised by frequent dystrophic calcification: a cross sectional study. Pediatric Rheumatology 12:1–5. 24397895 10.1186/1546-0096-12-2 PMC3896965

[3] Adelowo, O. , M. Nwankwo , and H. Olaosebikan . 2014. Juvenile dermatomyositis in a Nigerian girl. BMJ Case Reports doi:10.1136/bcr-2013-202132. PMC3987257 24706700

[4] Mandengue, C. E. , C. Nouedoui , and P. J. A. Atangana . 2012. Unrecognized juvenile dermatomyositis complicated by calcinosis universalis: a case report from Cameroon. Medicine et Sante Tropicales 23:458–461. 10.1684/mst.2013.0248 24401174

[5] Ntusi, N. B. , and J. M. Heckmann . 2010. Myopathy with a normal creatine kinase level in juvenile myopathic dermatomyositis. South African Medical Journal 100:25–5. 20429482

[6] Bohan, A. , and J. B. Peter . 1975. Polymyositis and dermatomyositis. New England Journal of Medicine 292:344–347. 1090839 10.1056/NEJM197502132920706

[7] Brown, V. E. , C. A. Pilkington , B. M. Feldman , and J. E. Davidson ; Network for juvenile dermatomyositis, paediatric rheumatology European society . 2006. An international consensus survey of the diagnostic criteria for juvenile dermatomyositis. Rheumatology 45:990–993. 16467366 10.1093/rheumatology/kel025

[8] Ramanan, A. V. , and B. M. Feldman . 2002. Clinical features and outcomes of juvenile dermatomyositis and other childhood onset myositis syndromes. Rheumatic Diseases Clinics of North America 28:833–857. 12506775 10.1016/s0889-857x(02)00024-8

[9] Fujimoto, M. , R. Watanabe , Y. Ishitsuka , and N. Okiyama . 2016. Recent advances in dermatomyositis‐specific autoantibodies. Current Opinion in Rheumatology 28:636–644. 27533321 10.1097/BOR.0000000000000329

[10] Feldman, B. M. , L. G. Rider , A. M. Reed , and L. M. Pachman . 2008. Juvenile dermatomyositis and other idiopathic inflammatory myopathies of childhood. Lancet 371:2201–2212. 18586175 10.1016/S0140-6736(08)60955-1

[11] Pachman, L. M. , K. Abott , J. M. Sinacore , et al. 2006. Duration of illness is an important variable for untreated children with juvenile dermatomyositis. Journal of Pediatrics 148:247–253. 16492437 10.1016/j.jpeds.2005.10.032

[12] Longo, D. L. , A. S. Fauci , D. L. Kasper , S. L. Hauser , J. L. Jameson , and J. Loscalzo . 2012. Paget's disease and other dysplasias of bone. P. 3144. Harrison's principles of internal medicine, vol. 2, 18th edition. McGraw‐Hill Companies, New York.

[13] Harper, J. , A. Oranje , N. Prose . 2000. Calcification and ossification in the skin. Pp. 783–784. Textbook of pediatric dermatology, vol 1. Blackwell Science, Oxford.

[14] Kobayashi, I. , M. Yamada , Y. Takahashi , et al. 2003. Interstitial lung disease associated with juvenile dermatomyositis: clinical features and efficacy of cyclosporine A. Rheumatology 43:371–374. 10.1093/rheumatology/keg040 12595639

